# Primary Sellar Neuroblastoma Masquerading as a Pituitary Macroadenoma

**DOI:** 10.1210/jcemcr/luad081

**Published:** 2023-07-12

**Authors:** Nandini Seshan, Simon Hanft, Gayotri Goswami, Arjun Syal

**Affiliations:** Department of Endocrinology, Department of Neurosurgery, Westchester Medical Center, Valhalla, NY 10595, USA; Department of Endocrinology, Department of Neurosurgery, Westchester Medical Center, Valhalla, NY 10595, USA; Department of Endocrinology, Department of Neurosurgery, Westchester Medical Center, Valhalla, NY 10595, USA; Department of Endocrinology, Department of Neurosurgery, Westchester Medical Center, Valhalla, NY 10595, USA

**Keywords:** esthesioneuroblastoma, primary sellar neuroblastoma, transsphenoidal resection, craniotomy

## Abstract

Olfactory neuroblastomas, or esthesioneuroblastomas, are rare and aggressive malignant tumors that typically arise from the olfactory neuroepithelium in the upper nasal cavity. In rare instances, they can be ectopic originating from areas outside the upper nasal cavity such as the sellar region. These tumors, also known as primary sellar neuroblastomas, may be mistaken for pituitary macroadenomas. We present a rare case of a primary sellar neuroblastoma in a 30-year-old woman with a prior diagnosis of presumed prolactinoma, status post transsphenoidal resection, with residual visual deficits, who presented with worsening vision and headaches. Pituitary magnetic resonance imaging showed a large sellar mass causing compression of the optic chiasm, and invasion of the right cavernous sinus and bilateral cavernous internal carotid arteries. The patient underwent a second transsphenoidal resection. Postoperatively, she developed central adrenal insufficiency, central hypothyroidism, central hypogonadism, and transient syndrome of inappropriate antidiuretic hormone secretion. Owing to rapid tumor regrowth, she underwent a craniotomy with plans for radiation treatment. This condition is challenging to diagnose and has poorly defined clinical management guidelines. An early, aggressive approach with surgical intervention is recommended.

## Introduction

Olfactory neuroblastoma, or esthesioneuroblastoma, is a malignant tumor that originates from the olfactory neuroepithelium in the upper nasal cavity with extension into the cribriform plate of the ethmoid sinus, anterior skull base, and orbit [1]. Ectopic primary olfactory neuroblastoma is extremely rare and arises from olfactory epithelium outside the upper nasal cavity including the nasopharynx, maxillary sinus, sphenoid sinus, optic pathways, and sellar region [2]. There are fewer than 40 reported cases of ectopic primary olfactory neuroblastomas and 11 reported cases of primary sellar neuroblastomas, of which 2 patient cases developed panhypopituitarism after transsphenoidal resection [3, 4]. We present a unique case of an ectopic primary olfactory neuroblastoma presenting as a sellar mass mimicking a pituitary adenoma.

## Case Presentation

A 30-year-old woman with a past medical history of a pituitary macroadenoma status post transsphenoidal resection with residual left eye blindness and right peripheral visual deficits presented with progressive vision loss and headaches. Two years prior she was receiving care outside the United States and was reported to have an elevated prolactin level, galactorrhea, and amenorrhea with improvement in prolactin levels after cabergoline initiation. A year later, she presented with bilateral peripheral vision loss leading to a car accident. Computed tomography and magnetic resonance imaging (MRI) showed a pituitary macroadenoma with mass effect on the optic chiasm with right cavernous sinus invasion and encasement of bilateral cavernous internal carotid arteries. She underwent her first endoscopic transnasal transsphenoidal surgery with incomplete resection. Pathology from initial surgery was consistent with nonfunctioning pituitary adenoma. Postoperatively, prolactin levels normalized and cabergoline was discontinued. Owing to frequent headaches and worsening vision including left-eye blindness and right peripheral visual field loss, she traveled to the United States for further evaluation. On presentation, she reported a 30-kg weight gain over 6 months, hair loss, constipation, and amenorrhea. Review of systems were negative for epistaxis, dizziness, galactorrhea, tremors, palpitations, easy bruising, purple striae, and abdominal pain. Physical examination was significant for complete left-eye blindness and right-sided peripheral visual field deficits. Repeat MRI showed substantial regrowth of the tumor.

## Diagnostic Assessment

Initial workup outside the United States included an elevated serum prolactin at 59.71 ng/mL (59.71 μg/L; reference range, 1.2-29.9 μg/L), while on cabergoline. Initial pituitary MRI showed a 3.7 × 3.1 × 3.2 cm homogeneously enhancing mass in the sella with suprasellar extension, compression of optic nerves, and chiasma suggestive of a pituitary macroadenoma. There was extension into the right cavernous sinus and encasement of bilateral cavernous internal carotid arteries. Initial transsphenoidal resection performed was incomplete because of difficulty in resecting the tumor. The pathology report of the initial resection demonstrated a nonfunctioning pituitary adenoma. Postoperatively, serum prolactin normalized and cabergoline was discontinued. However, within a few months prolactin levels rose to 42 ng/mL (42 μg/L), with associated worsening headaches and visual deficits.

**Figure 1.**
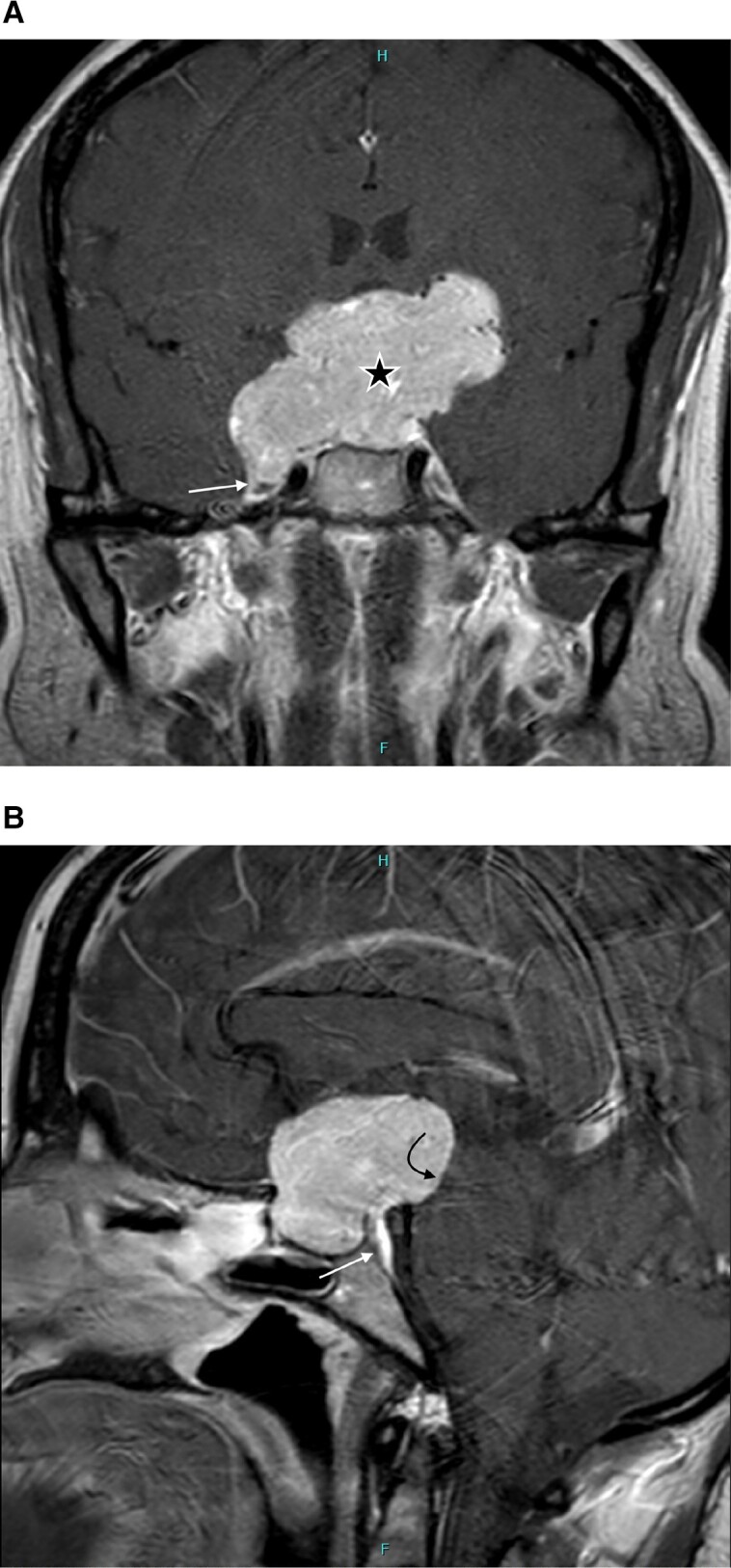
A, Coronal postcontrast image demonstrates the large, homogeneously enhancing sellar/supracellar mass with marked extension into the adjacent structures (star) There is extension into the right Meckel cave (arrow). B, Sagittal postcontrast image demonstrates extension of the mass along the dorsal clivus (arrow) as well as effacement of the interpeduncular cistern with mass effect on the midbrain (curved arrow).

**Figure 2.**
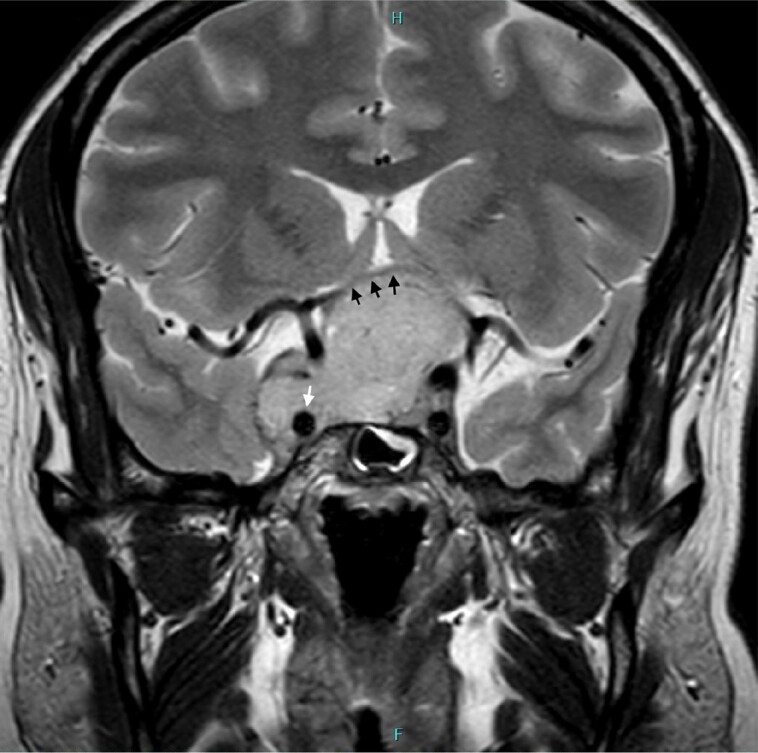
Coronal T2-weighted image again shows the sellar neoplasm with marked mass effect and thinning of the optic chiasm (arrowheads). There is near complete encasement of the right cavernous internal carotid artery (arrow).

In the United States, preoperative laboratory tests showed normal sodium, free thyroxine, estradiol, morning cortisol levels, and mildly elevated prolactin. The mildly elevated prolactin levels during the initial workup outside the United States and currently were likely due to stalk effect from the tumor. Pituitary MRI showed extensive suprasellar extension of a T2-hyperintense, homogenously enhancing, multilobulated sellar mass measuring approximately 4.3 × 3.5 × 6.2 cm (anteroposterior × craniocaudal × transverse) (Fig. 1A and 1B). The mass expanded the sella with extension into the middle cranial fossae and interpeduncular cistern. The mass extended along the dorsal clivus (see Fig. 1B) and invaded Meckel cave on the right (see Fig. 1A). There was marked mass effect on the prechiasmatic optic nerves and optic chiasm, with thinning of the optic chiasm. There was right greater than left encasement of the cavernous internal carotid arteries without evidence of occlusion (Fig. 2).

## Treatment

Progressive vision loss and persistent headaches in conjunction with imaging studies confirmed progressive growth of the preexisting tumor. The patient underwent the second transsphenoidal resection of the previously diagnosed sellar mass. The location and characteristic of the tumor including significant extension into the right cavernous sinus and encasement of the bilateral cavernous internal carotid arteries resulted in 80% to 90% resection of the mass and significant resection of the intrasellar portion. The lateral extent of the mass and its thick capsule did not lend itself to complete endoscopic resection. Histopathological diagnosis was esthesioneuroblastoma, a low-grade lesion, Hyams 1. Immunohistochemical staining was positive for chromogranin and synaptophysin. The proliferative index, Ki-67, was less than 1%. Tissue was immunonegative to neurofilament protein. After both resections of the same tumor, the pathology after the initial surgery outside the United States did not match the present pathologic diagnosis because of the absence of olfactory tissue.

Postoperatively, laboratory studies were remarkable for mild hyponatremia of 132 mEq/L (132 mmol/L; reference range, 135-145 mmol/L), low morning cortisol of 1.9 μg/dL (52.421 nmol/L; reference range, 3.7-19.4 nmol/L), and low free thyroxine 0.6 ng/dL (7.72 pmol/L; reference range, 0.7-1.9 pmol/L), indicative of the development of central adrenal insufficiency and central hypothyroidism, respectively. Total daily replacement of hydrocortisone was 30 mg, and levothyroxine 75 mcg daily was initiated.

One week postoperatively, mild hyponatremia persisted suggestive of syndrome of inappropriate antidiuretic hormone (SIADH) that was responsive to fluid restriction. She developed central hypogonadism with subsequent initiation of combined oral contraceptives. Bilateral vision significantly improved 3 months postoperatively.

Nine months later, repeat MRI showed rapid tumor regrowth in the surgical cavity. A pterional craniotomy was performed to decompress the optic nerve along the tuberculum sella. A gross total resection was not possible because of invasion of the right cavernous sinus and adhesions between the tumor, optic nerve, and adjacent deep-brain structures. The goal of this resection was to reflect enough tumor away from the optic nerves and chiasm to best preserve vision and optimal radiation planning.

## Outcome and Follow-up

The patient was continued on hormone replacement for central adrenal insufficiency, central hypothyroidism, and central hypogonadism. One month postoperatively, she continued to have complete left-eye blindness with improvement in right-sided vision. Owing to persistent tumor growth, the patient will undergo radiation and possible chemotherapy.

## Discussion

Olfactory neuroblastomas are rare, malignant tumors that originate from the olfactory neuroepithelium in the upper nasal cavity near the cribriform plate. They tend to invade adjacent tissues, metastasize, and cause cervical lymphadenopathy [5]. It affects men and women equally with a bimodal distribution between ages 20 and 50 years but can present at any age. Most common clinical symptoms include epistaxis, nasal obstruction, sinusitis, headaches, visual field deficits (bitemporal hemianopsia or oculomotor nerve palsy), and anosmia. The symptoms depend on the location and degree of tumor extension [4, 6].

**Figure 3.**
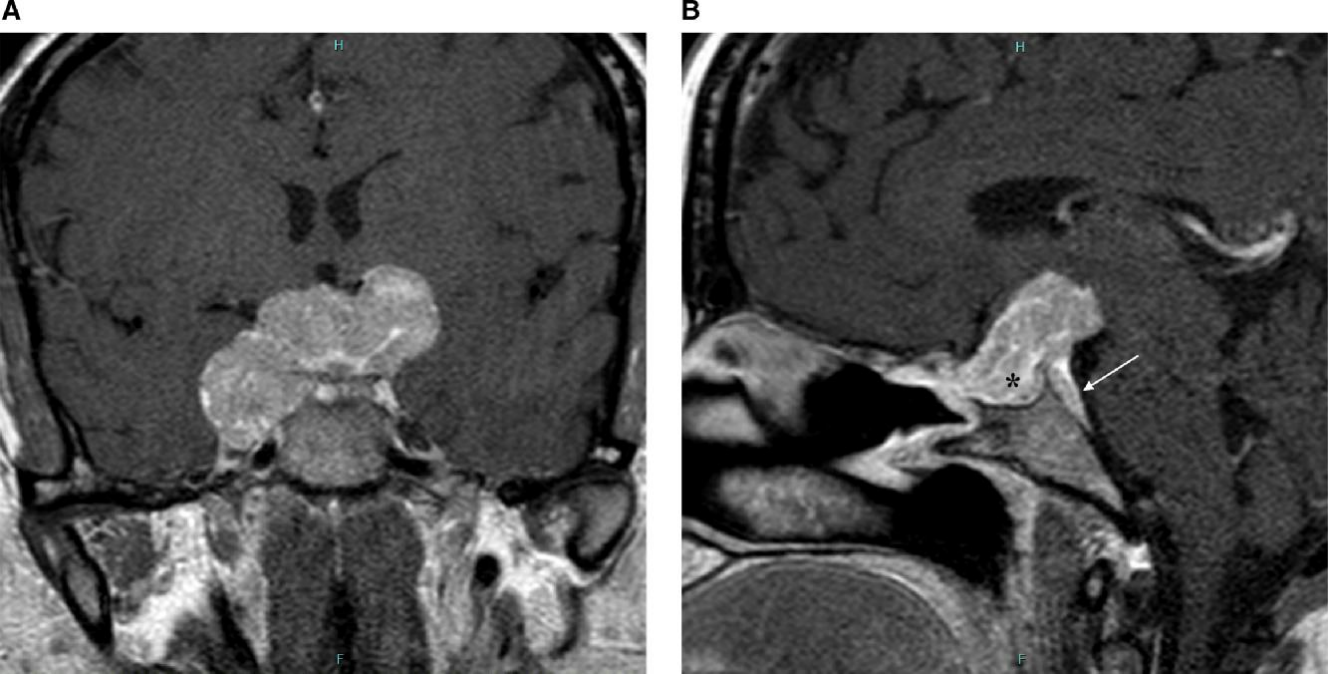
A, Coronal postcontrast image demonstrates a postoperative appearance of the enchanting cellar mass. B, Sagittal postcontrast images show the enhancing mass filling the resection cavity (asterisk) with persistent dorsal clinal extension (arrow).

Diagnosis is based on histology and immunohistochemistry. Histological appearance typically includes compact nests of small round cells with chromatin-rich nuclei and fibrillary background. Immunohistochemical staining is usually positive for neuroendocrine markers including chromogranin, synaptophysin, and neurofilament protein, while there is an absence of immunochemical staining for pituitary hormones (Fig. 3).

The histological grading system created by Hyams (I-IV) and local tumor extension appear to provide the most useful prognostic information. Two other clinical staging systems, TNM by Dulguerov et al and the modified Kadish classification, are also used to guide treatment. Due to the rarity of primary sellar neuroblastomas, these classifications systems have been used toward the staging and prognosis of ectopic olfactory neuroblastomas [4]. Hyams grading and Kadish staging systems are used to provide prognosis and help guide treatment decisions, including use of adjuvant therapies, such as radiation and chemotherapy [7, 9, 10].

Our patient presented with an ectopic olfactory neuroblastoma arising from the sella, outside the typical location of the upper nasal cavity, also known as a primary sellar neuroblastoma. These are extremely rare, with fewer than 40 cases reported, and challenging to diagnose because of resemblance to other types of tumors (craniopharyngioma, meningioma, paranasal cancer, and pituitary adenoma) [10].

Based on MRI, primary sellar neuroblastomas can be easily mistaken for a pituitary adenoma [9]. Radiologic imaging typically shows a large, compressive dumbbell-shaped sellar calcified mass, with extension into adjacent structures [4]. Endocrine disorders seen in sellar neuroblastomas were mainly hyperprolactinemia (6 cases) due to a pituitary stalk effect, gonadotropin insufficiency (4 cases), panhypopituitarism (2 cases), and SIADH (2 cases). All reported cases of primary sellar neuroblastomas had visual field deficits including oculomotor nerve palsy and bitemporal hemianopsia. The previously described characteristic symptoms along with neuroradiological features may facilitate inclusion of primary sellar neuroblastoma in the differential diagnosis [4, 6].

There are 11 cases of primary sellar neuroblastoma reported in the literature [2‐4, 6, 8, 10]. Roy et al [3] presented a case of a 44-year-old woman with hyperprolactinemia, bitemporal hemianopia, and a large sellar tumor with suprasellar and dural invasion. The patient underwent an incomplete surgical resection of the tumor, requiring hormone replacement and radiation therapy. Similar to our case, Mariani et al [9] described a case of a 35-year-old woman with amenorrhea and insomnia who was found to have hyperprolactinemia and a presumed pituitary macroadenoma. After initial treatment with cabergoline, 3 years later she developed partial bitemporal hemianopsia and repeat MRI showed strong progression of the tumor despite dopamine agonist therapy. The patient underwent transsphenoidal resection with complete resection of the tumor. Oyama et al [10] demonstrated a case of a 33-year-old man with headaches and bitemporal hemianopsia who was found to have a sellar mass with suprasellar extension, presumed to be a pituitary macroadenoma. The patient underwent transsphenoidal resection, and pathology showed a nonfunctioning pituitary adenoma. The tumor regrew 16 months later, resulting in subsequent craniotomy. Despite repeat resection and Gamma-Knife radiosurgery, the tumor rapidly regrew with cervical metastasis. He underwent 6 surgeries with subtotal resection of the tumor and subsequent panhypopituitarism.

Due to its rarity, there is no clear consensus regarding the management of primary sellar neuroblastomas. A combination of surgical resection followed by radiotherapy shows improved outcomes. The role of neoadjuvant chemotherapy remains controversial but shows promise in locally advanced or metastatic diseases. However, it has not been suggested as a treatment for primary sellar neuroblastomas [4‐6, 8, 9].

Our patient had a large ectopic olfactory neuroblastoma that originated in the sellar region and was mistaken for a pituitary macroadenoma and resulted in panhypopituitarism. Olfactory neuroblastomas have also been associated with paraneoplastic syndromes such as SIADH or ectopic adrenocorticotropin production [5]. Our patient developed transient SIADH postoperatively, which can occur after a transsphenoidal resection of pituitary macroadenomas.

In conclusion, primary sellar neuroblastomas are difficult to diagnose and can mimic pituitary macroadenomas. An early surgical approach combined with radiotherapy are crucial for appropriate and timely management. There is a high risk of recurrence with primary sellar neuroblastomas, reinforcing the need for close, long-term surveillance [6, 8, 9].

## Learning Points

- Primary sellar neuroblastomas are rare, aggressive tumors that arise from the olfactory epithelium outside the nasal cavity.
- Esthesioneuroblastomas can be mistaken for pituitary macroadenoma, especially if originating in the sellar region and result in panhypopituitarism especially after transsphenoidal resection.
- Diagnosis of primary sellar neuroblastomas is challenging, with no clearly defined clinical management guidelines.
- Management of primary sellar neuroblastoma include early aggressive intervention with surgery followed by adjuvant radiotherapy.

## Contributors

All authors made individual contributions to authorship: N.S., G.G., S.H., and A.S. were involved in the diagnosis and management of this patient; A.S., N.S., and G.G. were involved in manuscript submission; S.H. was responsible for surgical interventions. All authors reviewed and approved the final draft.

## Data Availability

Data sharing is not applicable to this article as no data sets were generated or analyzed during the current study.

## Abbreviations

MRI: magnetic resonance imaging
SIADH: syndrome of inappropriate antidiuretic hormone
